# Traditional Chinese medicine for gouty arthritis: A protocol for meta-analysis

**DOI:** 10.1097/MD.0000000000023699

**Published:** 2021-01-22

**Authors:** Meng-jun Pu, Cheng-jiao Yao, Li-ming Liu, Li-jun Ren, Yong-li Li, Yan Xie

**Affiliations:** a Department of Geriatrics of the Affiliated Hospital, North Sichuan Medical College, Nanchong; b Hospital of Chengdu University of Traditional Chinese Medicine, Chengdu, Sichuan, China.

**Keywords:** acupuncture, Chinese patent medicine, gouty arthritis, meta-analysis, protocol

## Abstract

**Background:**

Gouty arthritis (GA) is a chronic disease caused by monosodium urate crystal deposition. Repeated attacks of arthritis may lead to the deposition of urate to form gout stone, resulting in joint deformity and joint damage. Although GA is not fatal, it causes low work productivity and low quality of life. Western drug, such as febuxostat, colchicine, allopurinol, often cannot get satisfying curative effect, and may even lead to serious side effects, such as exfoliative dermatitis or uremia. However, the therapeutic effect of Traditional Chinese medicine is very satisfactory. The treatment effect of simiao powder, a Chinese patent medicine, combined with acupuncture was widely used on treatment of GA. Although it has been widely used in clinical practice, its relative effectiveness and safety have not been confirmed. Therefore, this study will use meta-analysis to verify the efficacy and safety of simiao powder combined with acupuncture in the treatment of GA.

**Methods:**

All randomized controlled trial of simiao powder combined with acupuncture for the treatment of RA from their inception 29 October, 2020 will be searched form the China National Knowledge Infrastructure, Wanfang Database, Chinese Science and Technology Periodical Database, Chinese Biomedical Literature Database, Pubmed, Embase, Web of Science, and the Cochrane library. Two authors will independently select studies, extract data based on pre-designed inclusion and exclusion criteria. Methodological quality assessment and risk of bias will be assessed using Cochrane bias risk tool. All data analysis will be conducted using Revman5.3, WinBUGS 1.4.3, and Stata14.2 software.

**Results:**

We will compare the different outcome indicators of various studies to provide a synthesis of the efficacy and safety of Simiao powder combined with acupuncture for GA patients. The main outcome measures included efficacy, remission rate (no drug symptoms), recurrence rate, clinical absolute score and relative score. Secondary outcome measures included related adverse reactions and uric acid concentration.

**Conclusion:**

The findings of the study will provide helpful evidence for the efficacy and safety of simiao powder combined with acupuncture in the treatment of GA.

**Registration number:**

This study protocol have been funded through a protocol registry. The registry number is INPLASY2020110028

## 1 Introduction

Gouty arthritisc (GA) is a chronic disease of deposition of monosodium urate crystals, which form in the presence of increased urate concentrations.^[1]^ Urate deposits in the joint, causing joint swelling, pain, movement disorders. The frequent attack of GA has seriously affected the life of patients and brought heavy burden to social medical treatment. The pathogenesis of GA is complex and it is not yet clear. It has been found that environment, genetics, immunity, diet, internal environment, trauma and stimulation are involved in the pathogenesis in varying degrees.^[2]^ Currently, western medicine treatment of GA include febuxostat, colchicine, allopurinol and so on. It can be divided into two categories: analgesics, uric acid lowering drugs.^[3]^ These drugs can temporarily reduce joint pain and uric acid level, but their side effects should not been ignored. For example, allopurinol may cause severe allergic reactions and fatal exfoliative dermatitis, especially in Asian population.^[4]^ Benzbromarone may cause severe renal damage, especially in patients with kidney stones.^[5]^ Therefore, we need to further explore new treatments methods for GA, with good efficacy and small side effects.

Traditional Chinese medicine (TCM) is an advanced experience that Chinese people have summed up in the struggle against diseases for thousands of years. Experienced doctors of Chinese medicine make choice of treatment drugs based on syndrome differentiation and a variety of clinical symptoms of patients. In TCM, GA is classified as “Bi zheng,” “Bai hu li feng jie,” and “Li jie bing.” The etiology of GA is summarized as abnormal of wind cold, dampness and phlegm stagnation. Among these, damp heat accumulation are regarded as the key factors of GA. TCM treatment includes oral Chinese herbal medicine, acupuncture, moxibustion, cupping, external application of Chinese medicine and other forms. In view of the cause of disease, TCM mostly adopts external treatment and internal treatment. Among them, the most widely used is acupuncture and Chinese herbal medicine simiao powder. Acupuncture is based on the theory of meridians, which stimulates characteristic acupuncture points to clear meridians and smooth the body's qi and blood.^[6,7]^ Simiao powder is composed of cortex phellodendri, atractylodes lancea, achyranthes bidentata and coix seed. It can clear away heat and damp, relax tendons and strengthen bones. Shi x et al. found that simiao powder can significantly improve the symptoms of acute arthritis in gout patients, its curative effect is not lower than colchicine, and the side effects are small.^[8]^ Another meta analysis reported that simiao powder are effective in the treatment of GA through anti-inflammation and lowering urate.^[9]^ Liu et al found that through acupuncture, the treatment of GA are invigorating spleen, tonifying kidney, dissipating dampness, resolving blood stasis, clearing away heat, removing toxic substances, soothing Liver, regulating qi, dredging collaterals and relieving pain.^[10]^ At present, simiao powder and acupuncture has been widely used due to low price, convenience, high efficacy, and few adverse reactions. The treatment of combined with internal and external treatment of TCM has also received good feedback from GA patients. Simiao powder combined with acupuncture is welcomed by patients in clinic.

Nonetheless, no systematic review or meta-analysis is available to assess the effect of simiao powder combined with acupuncture on treating GA. Therefore, the aim of this meta-analysis of randomized controlled trails is to evaluate the efficacy of simiao powder combined with acupuncture for GA, results in this study might prove to be more reliable for clinical practice and decision-making.

## 2 Methods

### 2.1 Protocol registration

This study protocol has been funded through a protocol registry on the INPLASY website (registration number is INPLASY2020110028). We will strictly abide by the requirements of the “the Preferred Reporting Items for Systematic Review and Meta-analysis Protocols” to report the meta-analysis.^[11]^ If there is any information adjustment during the entire study period, we will promptly correct and update it in the final report.

### 2.2 Inclusion and exclusion criteria

#### 2.2.1 Type of study

Randomized controlled trials including simiao powder combined with acupuncture for the treatment of GA will be included. Exclude non- randomized controlled trial (RCT), animal experiments, unclear results indicators such as images and other nonquantitative indicators. For the articles published repeatedly in Chinese and English journals, take the latest 1 published.

#### 2.2.2 Participants

Patients diagnosed with GA by ACR (1997), not restricted in age, gender, ethnicity, race, and disease stage. Reluctant to accept TCM treatment, patients with severe cardiovascular diseases, mental illnesses, Pregnant women, breast stage and cancer will be excluded.

#### 2.2.3 Interventions

##### 2.2.3.1 Experimental interventions

The intervention measures of the experimental group were simiao powder combined with acupuncture. It can be monotherapy or combination. RCT comparing the above 2 therapies can also be included, and those who combine Western medicine will be excluded.

##### 2.2.3.2 Control interventions

The control group received conventional treatment of western medicine, including the use of febuxostat, colchicine, allopurinol.

### 2.3 Outcome indicators

The main outcome indicators include effectiveness (recognized clinical efficacy evaluation criteria), effective including basic recovery, marked effect, improvement; remission rate (no drug symptoms), relapse rate, clinical absolute score, and relative score. Secondary outcome indicators: including any related adverse reactions,uric acid concentration.

### 2.4 Data sources and search strategies

We will search the following databases: the China National Knowledge Infrastructure, Wanfang Database, Chinese Science and Technology Periodical Database, Chinese Biomedical Literature Database, Pubmed, Embase, Web of Science, and the Cochrane library. Collect all the RCT on the treatment of GA with simiao powder combined with acupuncture. And manually search for references in related literature. The retrieval time is from the inception of the database to 29 October, 2020. The language is limited to Chinese and English. The search strategy is to combine search terms with subject words and free words. The primary selection process are shown in PubMed search strategy (Table 1).

**Table 1:** Search strategy used in PubMed database.

Number	Search terms
1	gouty arthritis
2	gout
3	arthrolithiasis
4	Bi zheng
5	Bai hu li feng jie
6	Li jie bing
7	1 or 2–6
8	Pharmacopuncture
9	acupuncture
10	8 or 9
11	Simiao powder
12	Simiao tang
13	Simiao pill
14	11or 12–13
15	Randomized Controlled Trials
16	Randomized Controlled Trial
17	RCT
18	controll clinical trial
19	Trials
20	Clinical Trials, Randomized
21	Trials, Randomized Clinical
22	Controlled Clinical Trials, Randomized
23	15 or 16–22
24	7 and 10 and 14 and 23

### 2.5 Selection of studies

**Figure 1:**
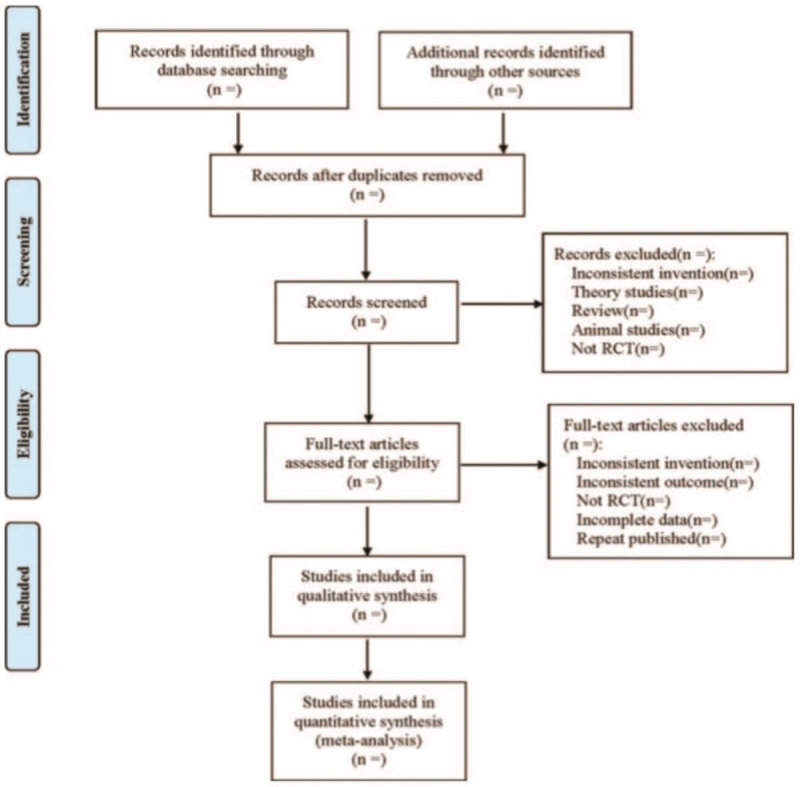
Flow diagram of study selection process.

Two authors independently complete the following process: according to the above search strategy to complete the process of document retrieval, import documents into EndNote X7 for centralized management. Then, according to the inclusion and exclusion criteria, filter the literature by reading the title and abstract. If it is not possible to determine whether the article meets the requirements based on the inclusion and exclusion criteria, then read the full text to select. In the entire literature screening process, if the 2 authors have different opinions, the third author joins the discussion to get a common opinion. The process of research selection is shown in Figure 1.

### 2.6 Data extraction

After the literature search process was completed, the 2 authors independently extracted the following information from the selected study: author, article title, year of publication, contact information, country/region, sample size, participants, diagnostic criteria, baseline characteristics, study design, random methods, blind methods, results, adverse events, and so on, and fill the extracted information into a pre-built Excel table. If necessary, we will contact the trial author for further information.

### 2.7 Dealing with missing data

If there is data loss in the included study, we will contact the original author of the article to obtain the original information. If the missing data is still not available, the existing data will be analyzed and a sensitivity analysis will be performed to address the potential impact of the missing data.

### 2.8 Risk of bias assessment and quality of selected studies

The 2 authors will independently assess the risk of bias (methodological quality) of the included studies based on the bias risk assessment tool recommended in the Cochrane “Risk of bias” assessment tool.^[12]^ Including 7 items: random sequence generation, allocation concealment, blind participants and personnel, blind assessment of results, incomplete result data, selective reports, and other biases. The results in each field will be divided into 3 levels: low bias risk, high bias risk, and unclear bias risk. The 2 authors will exchange assessment results and check whether the assessment results are consistent. If there is a disagreement, the third author will participate in the discussion and determine the final result.

### 2.9 Statistical analysis

Pairwise meta-analyses is conducted by RevMan5.3,^[13]^ Categorical data will be calculated with the risk ratio and 95% confidence intervals (95% CIs), continuous variables will be reported as mean differences or standardized mean differences with 95% CI. Heterogeneity will be evaluated by Chi-squared test and Higgins I^2^ test; If there is no obvious heterogeneity (*I*^2^ ≤50% and *P* > .10), the fixed effect model will be used; otherwise, the random effect model will be applied. Use WinBUGS 1.4.3 and Stata14.2 for network meta-analysis. In WinBUGS 1.4.3 software, Bayesian framework is implemented by the Markov chain Monte Carlo method,^[14]^ which is simulated by 4 chains, the number of iterations is set to 50000, and the step size is set to 10. At the same time, the Potential Scale Reduced Factor is used to evaluate the convergence of the results. When the PRSF value is approximately equal to 1.00, it indicates that the results are well converged, and the obtained results are highly reliable. If the PRSF is not within this interval, then continue to manually increase the number of iterations 50,000 times until the FRSF is within this range. In the case of many interventions involved, in the evidence network of each outcome indicator, the closed loop formed by the research with direct and indirect evidence needs to be tested for inconsistency. Calculate the inconsistency factor (IF), and judge whether there is inconsistency by the IF value and the P-value. If the IF is close to 0, the 95% CI starts at 0, and *P* > .05, it is considered that the direct comparison and the indirect comparison are consistent.^[15]^ At the same time, the node-split model is used to determine whether each node has local inconsistency. If *P* > .05, there is no obvious inconsistency. If there is no obvious inconsistency between the 2, the consistency model is adopted, otherwise, the inconsistency model is adopted. For the results obtained by the analysis of the consistency model, the stability of the results can be checked by the inconsistency model.^[16,17]^ Make evidence network diagram, correct-compare funnel diagram, and conduct inconsistency test in Stata14.2 software. Simultaneously calculate the value of surface under the cumulative ranking curves and the area under the surface under the cumulative ranking curves curve in order to rank the efficacy of various interventions. The value range is 0 to 100. The larger the value, the larger the area under the curve indicates the intervention and the greater the likelihood of being the best intervention.

### 2.10 Subgroup analysis and sensitivity analysis

If the Chi-squared test and Higgins *I*^2^ test detect obvious heterogeneity between studies, we will conduct a subgroup analysis from the following aspects: treatment time, GA classification, stage of disease, and so on. In order to ensure the Credibility of the research results, we will conduct a sensitivity analysis of the included literature and will eliminate low-quality literature.

### 2.11 Publication bias

If the included studies are sufficient (n≥10),^[18]^ the funnel plot will be used to assess the publication bias of the literature. If the funnel chart has poor symmetry, it indicates publication bias.

### 2.12 Assess the quality of evidence

The evaluation of the strength of the evidence will be based on the grading of recommendations assessment, development, and evaluation system, there are 4 levels of evidence strength: high, medium, low, or very low.

## 3 Discussion

In recent years, a large number of simiao powder or acupuncture in the treatment of GA have emerged. Wang indicate that the effects of needling the Shu, Yuan, and Mu acupoints can significantly reduce serum uric acid and xanthine oxidase levels compared with the western drug, possibly suggesting reduced renal damage.^[19]^ Another research found that acupuncture on 5 Shu in Spleen Meridian appeared to be safe and efficacious for decreasing serum uric acid in a Chinese HUA patient population. The mechanism might be associated with the decrease level of enzyme URAT-1.^[20]^ The findings of Lin suggested that simiao decoction was an effective therapeutic drug for GA and the gut ecosystem might act as a potential anti-inflammatory target of Simiao powder.^[21]^ Qiu found that simiao powder can significantly improve the symptoms and signs of GA and decrease the levels of UA and CRP.^[22]^ However, no reports of comparisons simiao powder and acupuncture on GA has been found. Therefore, this is the first meta-analysis to directly or indirectly compare the treatment of simiao powder and acupuncture in treating GA. It will provide the best evidence for clinical practice.

## Author contributions

**Conceptualization:** Li-ming Liu.

**Methodology:** Li-jun Ren.

**Project administration:** Yong-li Li.

**Software:** Yan Xie.

**Supervision:** Yan Xie.

**Writing – original draft:** Meng-jun Pu.

**Writing – review & editing:** Cheng-jiao Yao.
